# The Effect of Plasma-Treated Water on Microbial Growth and Biosynthesis of Gamma-Decalactones by *Yarrowia lipolytica* Yeast

**DOI:** 10.3390/ijms242015204

**Published:** 2023-10-15

**Authors:** Jolanta Małajowicz, Karen Khachatryan, Zdzisław Oszczęda, Piotr Karpiński, Agata Fabiszewska, Bartłomiej Zieniuk, Konrad Krysowaty

**Affiliations:** 1 Department of Chemistry, Institute of Food Sciences, Warsaw University of Life Sciences, Nowoursynowska Street 159C, 02-776 Warsaw, Poland; agata_fabiszewska@sggw.edu.pl (A.F.); bartlomiej_zieniuk@sggw.edu.pl (B.Z.); 2 Laboratory of Nanomaterials and Nanotechnology, Faculty of Food Technology, University of Agriculture in Cracow, Balicka Street 122, 30-149 Cracow, Poland; karen.khachatryan@urk.edu.pl; 3 Nantes Nanotechnological Systems, Dolne Młyny Street 21, 59-700 Bolesławiec, Poland; oszczeda@nantes.com.pl; 4 Faculty of Computer Science and Technology, Lomza State University of Applied Sciences, Akademicka Street 1, 18-400 Łomża, Poland; pkarpinski@ansl.edu.pl; 5 Faculty of Biology and Biotechnology, Warsaw University of Life Sciences, Nowoursynowska Street 159, 02-776 Warsaw, Poland; krysowatykonrad@gmail.com

**Keywords:** glow plasma, plasma-treated water, gamma-decalactones, lipases, β-oxidation, *Yarrowia lipolytica*

## Abstract

In recent years, the production of plasma-treated water (PTW) by low-temperature low-pressure glow plasma (LPGP) has been increasingly gaining in popularity. LPGP-treated water changes its physical and physiochemical properties compared to standard distilled water. In this study, a non-conventional lipolytic yeast species *Yarrowia lipolytica* was cultivated in culture media based on Nantes plasma water with heightened singlet oxygen content (Nantes PW) or in water treated with low-temperature, low-pressure glow plasma while in contact with air (PWTA) or nitrogen (PWTN). The research aimed to assess the influence of culture conditions on castor oil biotransformation to gamma-decalactone (GDL) and other secondary metabolites in media based on nanowater. The Nantes plasma water-based medium attained the highest concentration of gamma-decalactone (4.81 ± 0.51 g/L at 144 h of culture), maximum biomass concentration and biomass yield from the substrate. The amplified activity of lipases in the nanowater-based medium, in comparison to the control medium, is encouraging from the perspective of GDL biosynthesis, relying on the biotransformation of ricinoleic acid, which is the primary component of castor oil. Although lipid hydrolysis was enhanced, this step seemed not crucial for GDL concentration. Interestingly, the study validates the significance of oxygen in β-oxidation enzymes and its role in the bioconversion of ricinoleic acid to GDL and other lactones. Specifically, media with higher oxygen content (WPTA) and Nantes plasma water resulted in remarkably high concentrations of four lactones: gamma-decalactone, 3-hydroxy-gamma-decalactone, dec-2-en-4-olide and dec-3-en-4-olide.

## 1. Introduction

Cold plasma (CP) is a contemporary, emerging technology that modifies surfaces non-thermally without the use of chemicals and has minimal environmental impact. It can be generated by several sources, such as thermal energy, energy beam, and electric fields [1]. Systems used to activate cold plasma include micro-hollow cathode discharge (MCD), corona discharge (CD), gliding arc discharge (GAD), dielectric barrier discharge (DBD), plasma needle (PN) and atmospheric pressure plasma jet (APPJ) [2,3]. The formation methods of cold plasma impact its composition: specifically, the type and number of reactive forms and consequently also its technological applications. Due to its unique properties, which include low production costs and high effectiveness, it is extensively used in various fields such as waste management [4,5], air purification [6], the surface modification mainly of polymer materials [7], and the production/processing of nanoparticles [8,9]. Numerous publications detail and endorse the potential use of plasma for producing plasma-treated water (PTW) or plasma-activated water (PAW), which has antimicrobial properties due to the presence of reactive oxygen species (ROS), reactive nitrogen species (RNS) and hydrogen peroxide [10,11,12]. The combination of physical factors, such as low pH and high redox potential, with the formation of numerous active chemical compounds results in an antimicrobial effect.

Elkin et al. [13] and Reszke et al. [14] devised instruments for the plasma refinement of water by utilizing low-temperature and low-pressure glow plasma of low frequency (LPGP). The authors stated that this plasma-creating process gives liquids distinguished physical, chemical, biochemical and functional characteristics. LPGP avoids breaking valence bonds, so it does not trigger chemical reactions or create atomic oxygen radicals, hydrogen peroxide, and ozone [14,15]. Water treated with low-temperature, low-pressure glow plasma of low frequency (LPGP) is referred to as nanowater (NW) by the authors. Normally, water molecules form aggregates of up to 1000 units, but as a consequence of the cold plasma treatment, they are separated into individual, well-organized particles with a size of around 1 nM each. The dissociation of water macrostructure correlates with the concurrent excitation of triplet oxygen molecules that are dissolved in the water to the singlet state. Nanowater is characterized by low viscosity, very low density and high diffusivity. NW has a low dielectric constant, which facilitates the partial solution of non-polar compounds (such as lipids). It has been demonstrated that NW dissolves up to 35–40% more substances than traditional water. This facilitates the attainment of a higher concentration of nutrients or inorganic salts [13,14,15,16].

The advantages of utilizing NW have been demonstrated in several subsequent studies. For instance, the favorable impact of employing water treated with cold plasma in the cryopreservation of rams and boars’ sperm has been established. Additionally, irrigating wheat plantations with LPGP-treated water fostered plant development, resulting in greater yields [17]. A similar effect, associated with higher efficiency and improvement in the rate of seed germination and seedling development, was observed for Chinese cabbage watered with plasma water, the pH of which was neutralized by the immersion of metal ions (Mg^2+^ and Zn^2+^) [18]. Furthermore, besides stimulating the growth of herbs, NW also affected the essential oil composition. Watering a peppermint (*Mentha piperita rubescens*) crop in a greenhouse with LPGP-treated water over a period of 150 days resulted in a 24% increase in leaf growth and a 5% increase in stem growth. The extracts’ composition changed without affecting its antibacterial properties [19]. In the case of basil (*Ocinum bacilicum* L.), watering with LPGP-treated water positively impacted the seed germination rate. The application of LPGP under various conditions including contact with air (LPGPA), nitrogen (LPGPN), carbon dioxide (LPGPC) and methane (LPGPM) has been found to enhance the yield of essential oils by 40%, 60%, 20% and 20%, respectively [20]. Nanowater was evaluated for its potential as a catalyst for high-quality brewing malt production. Soaking spring barley grains in NW led to significantly higher water uptake and germination capacities. The resulting malt showed improved moisture content and seed index (1000-grain mass) [21]. Jaworska et al. [22] demonstrated that water exposed to low-temperature, low-pressure glow plasma of low frequency for 30 min stimulated the growth and pathogenicity of insecticidal fungi *Beauveria bassiana* and *Isaria fumosorosea*, which are used as biopesticides. Chwastowski et al. [23] conducted tests on NW to investigate its effects on the growth of microorganisms commonly found in the human body. The tests aimed to establish whether NW could stimulate or inhibit the growth of these microorganisms. The selected species, such as *Escherichia coli*, *Saccharomyces cerevisiae*, *Aspergillus niger*, *Candida albicans,* and *Enterococcus faecalis*, were monitored by measuring the optical density of their cultures. In the instance of culture in water treated with LPGP, a growth stimulation of approximately 20% was observed between the 12th and 24th hour, specifically for *E. coli* and *S. cerevisiae* cells.

The positive impact of NW in the aforementioned experiments, particularly in promoting microorganisms’ growth, has motivated further research into the potential use of this method to enhance the effectiveness of gamma-decalactone biosynthesis by *Yarrowia lipolytica* yeast cells. Gamma-decalactone (GDL), a cyclic ester of 4-hydroxydecanoic acid with a sweet peach fragrance, has the chemical formula C_10_H_18_O_2_. The biotechnological synthesis of β-oxidized ricinoleic acid through the biotransformation by yeast cells is a well-established process [24,25]. Despite the long history of research on GDL biosynthesis (the discovery of *C. tropicalis*’ ability to produce GDL dates back to 1963) [26], increasing the efficiency of the bioprocess remains a topic of ongoing scientific investigation [27,28].

The present article reports on a study that analyzed the growth and catalytic activity of *Y. lipolytica* yeast in the biotransformation of castor oil to GDL and other secondary metabolites using nanowater-based media. To the best of the authors’ knowledge, it is the initial attempt to assess the impact of NW on the potential of *Y. lipolytica* for producing GDL. In the experiments conducted, three types of treated water were used: water treated with LPGP in an atmosphere of nitrogen or air, and Nantes plasma water—a commercial product of NANTES Systemy Nanotechnologii Sp. z o.o. (Bolesławiec, Poland) company with a higher content of singlet oxygen.

## 2. Results

The study aimed to confirm the capability of *Yarrowia lipolytica* yeast cells to biosynthesize GDL and other volatile secondary metabolites when grown in liquid media composed of water treated with low-pressure, low-temperature glow plasma. In order to enhance the efficiency of the peach lactone biosynthesis reaction, this study analyzed various plasma water variants, including plasma water treated with nitrogen, air, and the commercial Nantes plasma water with heightened singlet oxygen levels. Plasma treatment resulted in oxygen or nitrogen enrichment, which may have a positive impact on increasing the aroma concentrations. The synthesis of GDL is reliant on the biotransformation reaction of castor oil, requiring its initial hydrolysis to ricinoleic acid and subsequent conversion into cyclic esters via the β-oxidation reaction cycle operating in yeast cell peroxisomes. Consequently, the research observed yeast cells growth together with their extracellular lipolytic activity.

### 2.1. Evaluation of Yarrowia lipolytica Yeast Cell Growth in Plasma Water-Based Media

**Figure 1:**
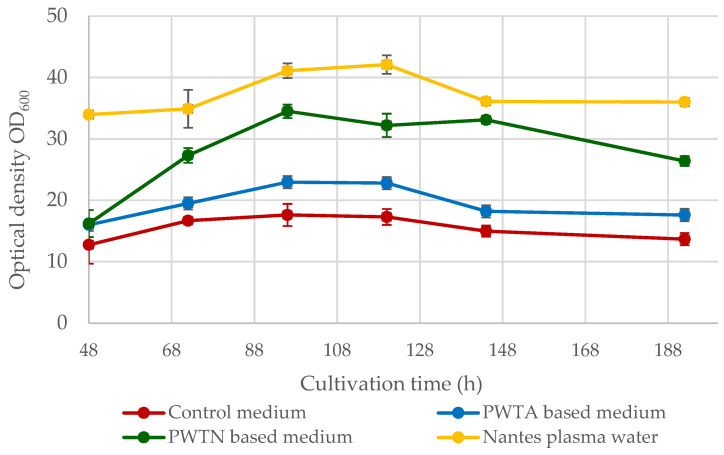
Growth curves of yeast *Yarrowia lipolytica* growing in the medium based on Nantes plasma water with heightened singlet oxygen level or on the water treated with low-temperature, low-pressure glow plasma in contact with air (PWTA) or nitrogen (PWTN).

This study confirms the effect of plasma water on the growth of *Yarrowia lipolytica* yeast cells, using the optical density (OD_600_) of the media and the yield of yeast cell dry matter as measures. Figure 1 illustrates the changes in the OD_600_ parameter over an 8-day culture period.

The OD_600_ values curves for the conducted cultures exhibit a similar course, peaking between 96 and 120 h of culture. The highest OD_600_ values were observed for yeast cultured in Nantes plasma water-based media (commercial water from Bolesławiec). At day 5 (120 h), the OD_600_ parameter reached approximately 42.1 ± 1.5. Slightly lower optical density values were observed for the culture that was based on plasma water in contact with nitrogen (PWTN) with a maximum of 34.5 ± 1.2 at a cultivation time of 96 h. The culture grown on plasma water in contact with air (PWTA) exhibited a considerably lower optical density of the medium, not exceeding the value OD_600_ = 25. Compared to the microbiological media used, which varied regarding the type of water employed, the control medium (prepared on distilled water) exhibited markedly lower optical density. The maximum value at 96 h was only 17.6 ± 1.8, which was twice as low as that in the Nantes water medium, showing the highest values.

**Figure 2:**
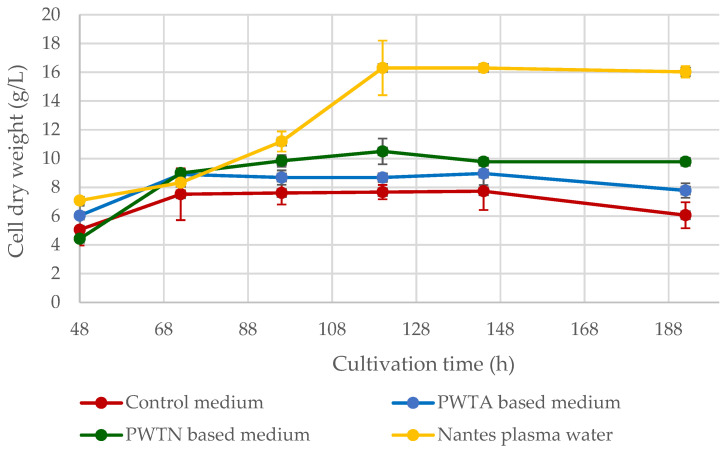
Dry cell mass of yeast *Y. lipolytica* cultivated in the medium based on Nantes plasma water with heightened singlet oxygen level or on the water treated with low-temperature, low-pressure glow plasma in contact with air (PWTA) or nitrogen (PWTN).

Additionally, the yeast cell dry mass was measured. Figure 2 illustrates the fluctuating yield of *Yarrowia* yeast cell dry matter over an 8-day culture.

The dry cell mass yield curves of yeast grown in plasma water-based media align with the optical density data exhibited in Figure 1. The best yeast growth parameters and highest biomass yield over the culture cycle were found when utilizing Nantes water with enhanced singlet oxygen content. On day 5 of the culture, the maximum biomass synthesis was 16.31 ± 0.91 g/L, which is approximately 9.5 g/L greater than the control culture. Cultures based on plasma water in contact with air or nitrogen resulted in higher biomass yields of approximately 2–4 g/L. It is worth noting that the use of plasma water in the media (regardless of the plasma treatment) increased the logarithmic growth phase of the microorganisms, as shown in Figure 1 and Figure 2. In the standard medium, the yeast reaches the stationary phase after roughly 55 h. In contrast, when using media containing plasma water in touch with nitrogen or air, the logarithmic growth phase continues for up to about 72–96 h, and it is even longer in the case of water containing an elevated amount of singlet oxygen.

To evaluate the effect of plasma treatment of the water utilized in microbiological media, specific kinetic parameters for distinct cultures were evaluated and are displayed in Table 1.

A comparison was conducted to assess the specific growth rate of the microorganisms, maximum biomass concentration and substrate biomass yield. Nantes plasma water yielded the highest values for all parameters. This culture’s maximum specific growth rate was roughly 1.6–2.2 times higher than PWTA and PWTN media, respectively, and approximately 2.4 times higher than the control medium. The maximum biomass concentration expressed as the number of colonies count was 0.55–0.88 higher in comparison to the media with plasma water under air or nitrogen or the control medium.

### 2.2. Effect of Plasma Water on the Lipolytic Activity of the Y. lipolytica Yeast

The extracellular lipolytic activity of the microorganism is crucial to the efficiency of the biosynthesis of GDL, a peach-aroma cyclic ester, and other volatile metabolites. The substrate in the synthesis of the above-mentioned lactone is ricinoleic acid, which constitutes about 90% of castor oil triglycerides [31], which are used in biotransformation media. The first step of biosynthesis is the hydrolysis of triglycerides catalyzed by lipases.

**Figure 3:**
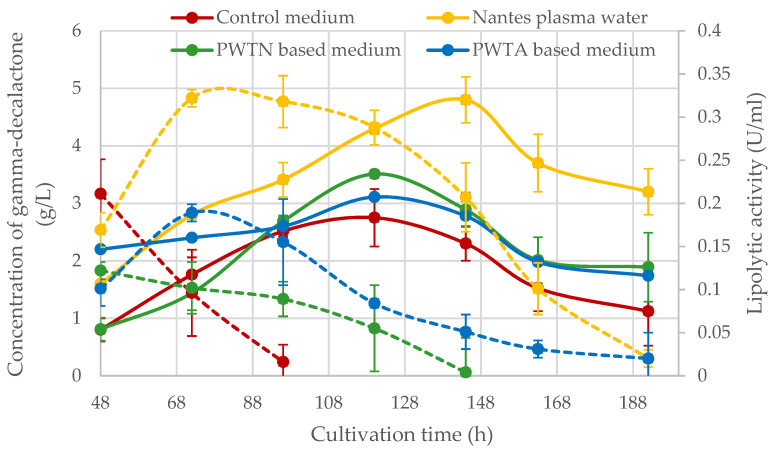
Kinetics of extracellular lipolytic activity (dashed lines) and production of gamma-decalactone (solid lines) in media based on low-pressure glow plasma water saturated with air (PWTA), nitrogen (PWTN) or molecular oxygen (Nantes plasma water).

Figure 3 shows data on the extracellular lipolytic activity of *Yarrowia* yeast grown on plasma water media (dashed lines). The results indicated that the highest lipolytic activity at the level of approx. 0.322 ± 0.013 U/mL was found in the medium based on Nantes plasma water with an increased singlet oxygen content. The maximum activity in the medium was observed on the third day of cultivation. A similar time of maximum activity was also noted for the PWTA medium, but this was approximately 1.7 times lower (approx. 0.189 ± 0.016 U/mL). The lipolytic activity curves determined for the PWTN medium and the control medium were slightly different. In both cases, the maximum activity occurred between 24 and 48 h of cultivation, which was not captured due to measurements taken from 48 h onwards. It is worth emphasizing that in the media based on plasma water, extracellular lipolytic activity was detected for a much longer period of culture than in the control medium. For cultures carried out in media based on plasma water enriched with singlet oxygen (Nantes PW) as well as PWTA medium, lipolytic activity was maintained throughout the culture period (192 h), while for the control medium, the lipase activity completely disappeared after 96 h, and no active lipases were detected.

**Figure 4:**
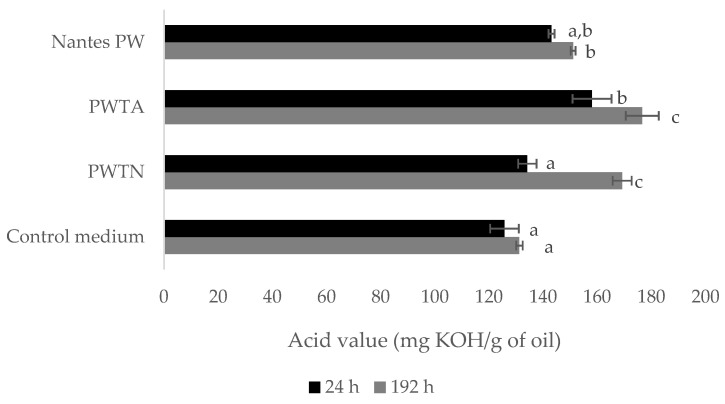
Acid value in media based on low-pressure glow plasma water saturated with air (PWTA), nitrogen (PWTN) or molecular oxygen (Nantes plasma water) with castor oil as a hydrophobic substrate. Mean values followed by the same letter are not significantly different according to HSD Tuk-ey (*p* ≤ 0.05).

The spectrophotometrically determined extracellular lipolytic activity of *Y. lipolytica* yeast in culture media was verified by determining the acid value in media (Figure 4). In each of the analyzed media, the parameter was high on the second day of culture and on the last day of the experiment. In the control medium, the acid value was approximately 125.7 ± 1.2 mg KOH/g on the 48th hour of culture and approximately 131.2 ± 5.3 mg KOH/g on the 8th day of culture (192 h). In the plasma water-based media, the acid value was statistically significantly higher, from 8 to 32 mg KOH/g (at 48 h of culture) and from 20 to 46 mg KOH/g oil (on 8th day of culture).

The determined acid value can be partly correlated with the activity of extracellular lipases, which carry out the hydrolysis of castor oil triacylglycerols. However, it should be taken into account that the ricinoleic acid released in the hydrolysis process is partially involved in the β-oxidation cycle and converted into GDL or other lactones. The determined acid value is reduced by the amounts of ricinoleic acid, which is involved in the yeast catabolic cycle.

**Figure 5:**
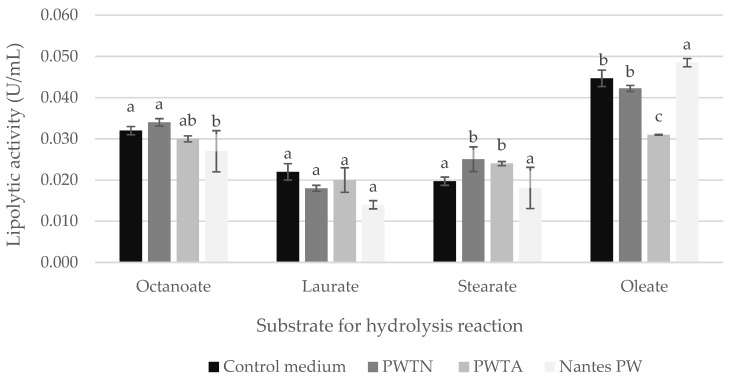
Lipolytic activity of extracellular native lipase YLLip2 verified in the context of the type of plasma water used in culture medium and the substrate used. Mean values followed by the same letter are not significantly different according to HSD Tukey (*p* ≤ 0.05).

Analysis of the kinetics of extracellular lipolytic activity (Figure 3) in combination with the determined acid value (Figure 4) resulted in the new hypothesis that plasma treatment under varying conditions of the water used in microbial cultures affects lipase production/secretion, or more meaningfully, affects the activity of the enzyme itself. To unequivocally answer this question, the lipolytic activity of the native enzyme YLLip2, isolated from the culture medium and purified by chromatography, was determined. In addition, tests were carried out with four substrates varying in carbon chain length *p*-nitrophenyl esters, using *p*-nitrophenyl octanoate, laurate, stearate and oleate. The results of this experiment are presented in Figure 5.

The data collected showed that there was no clear correlation between the plasma treatment of water and the activity of the native extracellular enzyme YLLip2. However, such a correlation was visible within the type of a substrate used. The length of the carbon chain of the *p*-nitrophenyl ester and the chain saturation had a significant impact on the catalytic activity of the enzyme. Lip2p showed the highest activity, irrespective of the type of water used in the culture medium, toward *p*-nitrophenol oleate (C18 monounsaturated acid). In the case of media based on commercial Nantes plasma water or PWTN, the activity toward this substrate was approximately 0.048 ± 0.002 U/mL and 0.042 ± 0.003 U/mL, respectively, while in the presence of *p*-nitrophenyl stearate (C18 saturated acid), the activity decreased to 0.018 ± 0.001 U/mL and 0.025 ± 0.003 U/mL, respectively. Therefore, it is clear that unsaturated fatty acids enhanced the catalytic activity of lipases.

The effect of carbon chain length becomes significantly apparent when comparing YLLip2 activity toward stearate (C18) and *p*-nitrophenyl octanoate (C8). In relation to octanoate, the activity was about 0.027 U/mL ± 0.001 U/mL for the reaction carried out in the presence of commercial Nantes plasma water, then 0.034 U/mL ± 0.001 U/mL using WPTN, and 0.032 ± 0.002 U/mL for the control substrate. When *p*-nitrophenyl stearate was used, the activities were 0.009 to 0.012 U/mL lower for each water variant. Hence, it can be concluded that the shorter carbon chain of the hydrolysis substrate affected the level of determined YLLip2 catalytic activity. It also confirmed that the studied enzyme was a “true” lipase.

### 2.3. Effect of Plasma-Treated Water on the Synthesis of Gamma-Decalatone and other Volatile Metabolites

The yeast *Y. lipolytica*, successfully synthesized the fragrance compound—gamma-decalactone (Figure 3—solid lines), regardless of the variant of plasma water used for their culture. The highest GDL concentration was achieved in the Nantes plasma water, and it amounted to approximately 4.81 ± 0.51 g/L at 144 h of culture. Lower concentrations of about 3.11 ± 0.03 g/L and 3.51 ± 0.01 g/L were obtained for the media PWTA and PWTN, respectively. In each nanowater-based media, the concentration of the peach-aroma compound was higher relative to the control culture, and this difference ranged from approx. 0.36 to 2.05 g/L, which as a percentage represented from 13% (for the PWTA medium) to 74% (for Nantes PW medium). Irrespective of the variant of culture carried out, the curves of the time-varying GDL concentration peaked at the 5th (control culture) or 6th (plasma water-based media) day of cultivation.

In order to compare the biotransformation yield of yeasts grown in plasma water-based media in the presence of individual gases, kinetic parameters such as the GDL accumulation efficiency in relation to biomass yield and substrate, as well as the average specific and volumetric accumulation rate of this compound, were also compared (Table 2).

In relation to biomass yield, the GDL accumulation efficiency for all plasma water types was lower compared to the control medium. The lowest value of 0.294 g/g_d.w._ was achieved when Nantes plasma water was used in the medium. This was approximately 18% lower than the control medium. The parameter of GDL accumulation calculated relative to the substrate showed an opposite trend. The medium with singlet oxygen-enhanced water, for which the Y_GDL_/S parameter was 0.048, stood out as statistically significant, while the medium PWTN or PWTA showed a lower value of this parameter by approximately 35%. Comparing the average volumetric rate of GDL accumulation (qLV_GDL_) in the different media, it can be seen that between the most efficient (Nantes PW) and the least efficient biotransformation (control medium), the volumetric rate was about 1.4 times lower. In the case of the average rate of specific accumulation of GDL related to the yeast cell dry matter yield (qL_GDL_), the specific production rate statistically differed by about 0.7 times in favor of the control medium.

**Figure 6:**
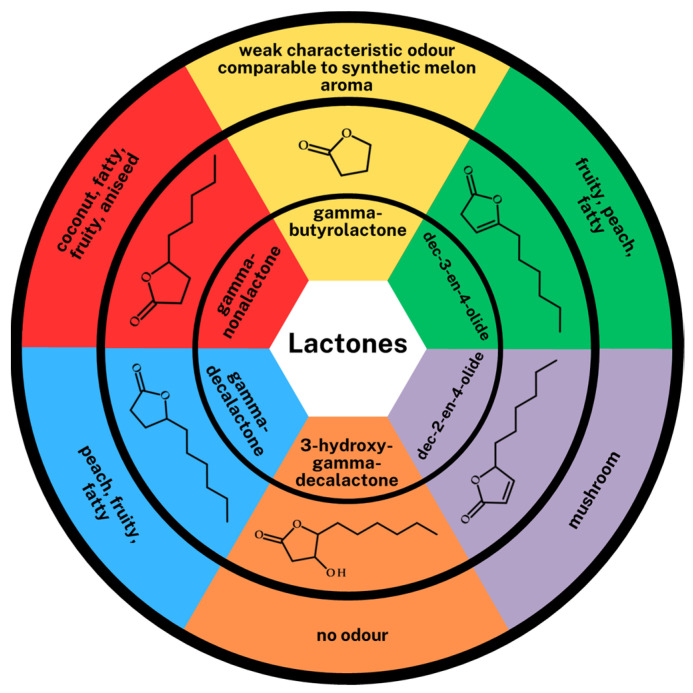
Lactones identified in biotransformation media along with their structure and sensory properties, which are given based on Dufossé et al. [32].

The analysis and identification of volatile compounds produced in the individual biotransformation medium was performed using the HS-SPME-GC-MS technique. During the analysis, special attention was focused on the group of lactones. Twenty-nine volatile compounds were detected in the samples from the four different biotransformation media with *Y. lipolytica* after 72 h of cultivation (Table 3). Regardless of the type of medium (based on distilled or plasma water), higher alcohols (3-methyl-butan-1-ol, *n*-heptanol, oct-1-en-3-ol, 2-phenylethanol), carbonyl compounds (aldehydes: hexanal, heptanal, benzaldehyde, phenylacetaldehyde and one ketone: 2-octanone), fatty acids (2-methyl-butanoic acid, 3-methyl-butanoic acid, 2-hydroxyoctanoic acid, 4-hydroxydecanoic acid, 3,4-dihydroxy-decanoic acid) and lactones (gamma-butyrolactone, gamma-nonalactone, gamma-decalactone, 3-hydroxy-gamma-decalactone, dec-2-en-4-olide, dec-3-en-4-olide) were detected in each sample. Interestingly, no esters were detected. In the group of volatile compounds, aldehydes showed the highest concentration, apart from the type of biotransformation medium, from 39.59 ± 0.86% (control medium) to 44.47 ± 3.15% (PWTN medium). Statistically significant differences can be noticed in the content of higher alcohols in the control medium (higher alcohol concentration) in relation to the media based on plasma waters. The difference is also visible in the concentration of lactones. It is significantly higher (nearly twice) in media with PWTA or Nantes plasma water, 22.03 ± 0.87% and 24.55 ± 0.36%, respectively, relative to the control medium—13.01 ± 0.44%. In media with higher oxygen content (PWTA) and Nantes plasma water, particularly high concentrations for four lactones, i.e., gamma-decalactone, 3-hydroxy-gamma-decalactone, dec-2-en-4-olide and dec-3-en-4-olide, were observed. The structures and sensory properties of all lactones identified in the media are shown in Figure 6. The structures of the above-mentioned lactones are closely related to those of GDL, but their sensorial properties are quite different.

## 3. Discussion

The aim of this study was to investigate the effect of plasma water on the growth and catalytic activity of *Y. lipolytica* yeast in the reactions of castor oil biotransformation to GDL and other metabolites. The research presented in the article was carried out using plasma water, under an air or nitrogen atmosphere, in a reactor patented by Elkin et al. [13] or commercially available Nantes plasma water (Bolesławiec, Poland) with the composition given in Section 4.2.3 with increased singlet oxygen content.

According to literature data, water treated with low-temperature low-pressure plasma differs in physical and chemical parameters in relation to standard distilled water. These differences concern, e.g., water pH, conductivity, surface tension or the amount of oxygen dissolved in water [15]. The analyses of plasma water in different gas atmospheres conducted so far have shown that regardless of the gas environment in which the plasma is performed, the water macrostructure changes. It undergoes declustering, creating clathrates that are energetically stable and lose the ability to form gigaclusters [33]. However, the level of changes in the structure and physicochemical properties of plasma water depends on the plasma generation parameters and the time of its action on the water. The water used in our experiments was obtained as a result of the action of low-pressure, low-temperature, low-frequency glow plasma, under a pressure of 5 × 10^−3^ mbar, voltage of 600 V, 50 mA and frequency of 10 KHz [14]. The parameters of this treatment cause declustering of the water macrostructure with the simultaneous excitation of triplet oxygen molecules dissolved in water to the singlet state. Raman spectra indicate that singlet oxygen molecules are found inside plasma water clathrates [15]. It is also worth noting that in contrast to the commonly known plasma methods, plasma under reduced pressure, with the parameters described above, did not generate ozone or hydrogen peroxide in water, which are considered to be strong antimicrobial oxidizing agents [34]. Ozone participates in the oxidation of sulfhydryl groups, which are quite numerous in enzymatic proteins of microorganisms [35], while hydrogen peroxide generates hydroxyl radical OH● intracellularly, which through the Fenton or Haber–Weiss reaction contributes to oxidative damage, stimulating the breakdown of the double helix of the DNA strand [36,37].

The effect of using plasma water in a medium on the growth of *Y. lipolytica* yeast cells was analyzed in the first stage of the research. In the aspect of GDL biosynthesis, it was important to verify to what extent plasma water stimulates or inhibits the growth of these microorganisms. The results of the measurements of the optical density OD_600_ (Figure 1) and the yield of dry cell mass (Figure 2) showed that the use of plasma water contributes to the increase in the yield of yeast cell biomass and extends the duration of the stationary phase compared to the control medium (in which distilled water was used). The positive effect of plasma water was observed regardless of the gas atmosphere in which the water was treated by plasma. Clearly, the best results were obtained with the use of commercial Nantes plasma water. It was characterized by an increased content of singlet oxygen. This was due to passing pure (100%) O_2_ through the water. The oxygen content in Nantes water is around 8.13 mg/L, and the singlet oxygen content is around 10–12 mg/L. Compared to traditional water, this value is higher by approx. 2 mg/L; at a temperature of 25 °C and a pressure of 101.3 kPa, water contains approx. 6.04 mg/L of oxygen [38]. This higher concentration of oxygen content may be a key factor in increasing cell concentration in the medium. The use of oxygen-enriched air has become a common strategy for the production of various biological substances by means of whole-cell catalysis. Baez and Shiloach [39] described a method for increasing cell density and improving process efficiency by supporting the aerobic growth of eukaryotic microorganisms in submerged fermentation bioreactors. Try et al. [29] also indicated the beneficial effect of oxygen on the growth of *Y. lipolytica*. The concentration of 30% oxygen in the medium was favorable for the growth of the yeast, which reached the stationary phase earlier compared to the other conditions used. The cultures at the initial oxygen ratio of 20% and 40% later reached the stationary phase similar to the results of the present study. The positive effect of oxygen on the production of *Y. lipolytica* biomass is also confirmed by Lopes et al. [40]. The increase in oxygen availability caused by increasing the air pressure to 6 bar had a clear positive effect on yeast metabolism, and the biomass yield increased by 67.8% and 86.4%, respectively, compared to with experiments at atmospheric pressure (control). Other unconventional yeasts, such as *Pichia pastoris* or *Kluyveromyces marxianus*, were also characterized by higher biomass yields at higher oxygen concentrations in the medium [41].

Taking into account kinetic parameters such as the maximum specific growth rate (μ_max_), maximum biomass concentration and level of yield of biomass from the substrate (Table 1), relatively high values of these parameters were also achieved for medium based on plasma water treated under nitrogen. Higher values of the yield of yeast biomass growing on the medium enriched with nitrogen (compared to the control) are confirmed in the literature. Hapeta et al. [42] emphasized that more nitrogen in the medium promotes rapid cell division and generates higher biomass yield. The availability of nitrogen also affects the level of substrate utilization by *Y. lipolytica*, but there are some differences in the way it is assimilated: co-utilization or sequential. It is hypothesized that this phenomenon may be related to carbon and nitrogen source metabolism. At higher nitrogen concentrations (when the C/N ratio is about 20), the cellular machinery of *Y. lipolytica* is geared toward biomass production. While nitrogen becomes scarce (C/N 40), cells begin to store available carbon in storage molecules such as triacylglycerols and glycogen. The research also confirms that nitrogen availability has a major impact on gene expression in chemostat cultures [43].

It should be remembered that the structure of plasma water and the state of dissolved gases have an impact on living organisms. Water treated with plasma, after passing air or nitrogen through it, acquires different physicochemical properties [15]. In a natural macrosystem, water molecules form a network of hydrogen bonds with structured clusters. Molecules can arrange themselves in various structures, e.g., small four-molecule structures, which can then be grouped into relatively stable octamers [33]. Water molecules also form much larger clusters of water capable of merging and moving through space. These clusters can interconvert between lower and higher structures. The balance between these structures and subsequent changes in the properties of water can be affected by the presence of dissolved gases and components of microbial media and temperature [44,45]. Gaseous nitrogen and oxygen differ slightly in their molecular properties, but the level of solubility of oxygen in water is twice as high as that of nitrogen [33]. Oxygen molecules interact with the aquatic environment and, according to ab initio molecular dynamics simulations, can form two types of clusters: with and without hydrogen bonding [46]. The second kind of the structural organization of the O_2_-water hydration complex is more preferable energetically. Under plasma treatment, oxygen molecules can be converted to a singlet state and nitrogen molecules can be converted to a triplet excited state. Molecules reside in water clathrates [47].

Growing in medium based on plasma water, enriched with oxygen or nitrogen, can cause the physiological response of microorganisms in which they transition from normoxia to hyperoxia. Furthermore, it may cause the deactivation of some genes with the simultaneous activation of protective genes involved in redox homeostasis and oxidative stress. In an environment with a higher concentration of oxygen or nitrogen, there is an increased accumulation of intracellular reactive oxygen and nitrogen species (RONS) that can reach a harmful level and overwhelm the defense and repair mechanisms of cells [17]. Reactive species can change protein structures, leading to enzyme inactivation. Tolouie et al. [48] indicated, e.g., the inactivation of lipoxygenase and lipase in wheat germ by treating them with cold plasma. However, it should be remembered that RONS can cause both deactivating and activating effects, depending on their concentration [49]. In plasma water, RONS concentrations can be controlled by the plasma activation time, feed gas composition, applied voltage, flow rate and plasma application method [50]. The positive effect of plasma on both the viability of microorganisms and the production of enzymes by them has been described in several studies [51,52,53,54]. Also, the results of our analyses showed that the cultivation of microorganisms in a medium with plasma water contributes to the higher activity of lipases (Figure 3). Higher values of the acid number in media based on plasma waters could be also noticed (Figure 4). However, it should be borne in mind that the acid number, expressed as the amount of mg of KOH used per 1 g of the lipid fraction extracted from the medium, does not fully reflect the level of triglyceride hydrolysis. In biotransformation reactions aimed at the synthesis of GDL with the use of the substrate, i.e., castor oil, released as a result of hydrolysis, ricinoleic acid is included in situ in the β-oxidation cycle, leading to the production of gamma-lactones. Thus, the determined acid number is the resultant of the lipolytic activity and the activity of the β-oxidation process taking place in the peroxisomes of yeast cells. The positive effect of the atmospheric pressure cold plasma treatment of *P. pastoris* cells on the increase in the amount of protein and the activity of the phytase enzyme in culture media is also confirmed by Farasat et al. [51]. According to the authors, the higher activity of enzymes after plasma treatment may result from a slight development of the tertiary structure of the enzyme. Veerana et al. [54], however, described that a certain level of oxidation reduction potential of the medium, which increases due to the action of plasma, may be a factor causing enzyme activation or higher secretion.

Since the higher catalytic activity may be the result of increased secretion of the enzyme protein or increased activity of the enzyme itself, or a synergistic effect of both aspects, the effect of plasma water on the activity of the isolated YLLip 2 enzyme was also tested in this study. YLLip 2 is a glycosylated triacylglycerol hydrolase with a molecular weight of 38 kDa and a length of 301 amino acids, which is responsible for all the extracellular activity and encoded by the lipase 2 gene [55]. The activity of YLLip 2 was measured using substrates with different lengths of the C_8_–C_18_ carbon chain, which were additionally differentiated by the level of their unsaturation. The experiment did not show an unequivocal correlation between the water plasma treatment and the activity of the native extracellular YLLip 2 enzyme. However, the relationship between the level of YLLip 2 activity and the type of substrate used was revealed. The length of the *p*-nitrophenyl ester carbon chain and the saturation of the chain have a significant impact on the catalytic activity of the native enzyme. In the case of lipases from filamentous fungi, the chain length specificity generally varies between C_8_ and C_18_ [3,56,57]. Takó et al. [57] confirmed that long-chain fatty acids generally reduced enzyme activity in a dose-dependent manner. Medium-chain acyl groups activated lipases by the specific or cooperative binding of fatty acids on the protein surface. It can be concluded that the higher extracellular lipolytic activity of yeast grown in media based on plasma water is the result of a higher secretion of lipases rather than a higher activity of the enzyme itself. The higher concentration of lipases in the media with nanowater may also result from the higher concentration of yeast cells (Figure 2).

Analyzing the data from Figure 3, it can be seen that the kinetics of lipase activity in media with a higher oxygen content is slightly different in relation to the medium enriched with nitrogen or the control medium. In aerobic media, the maximum lipolytic activity was observed on the 3rd day of multiplication, while in nitrogen media, the maximum occurred up to 48 h. The extracellular kinetic profile of lipase activity reported by Braga et al. [58] and Lopes et al. [40] is consistent with our observations and shows that *Y. lipolytic* activity reaches a maximum and then decreases. Lipolytic activity in the culture medium is the result of a balance between the production of lipases and their degradation caused by the action of proteolytic enzymes [59]. Perhaps the secretion profile of proteases is varied in media differentiated by oxygen and nitrogen concentrations.

Significantly higher activity of lipases in the nanowater-based medium (compared to the control medium) was promising in the context of GDL biosynthesis based on the biotransformation of ricinoleic acid, which is the main component of castor oil (used as a substrate). The catabolism of hydroxylated fatty acids by yeast has been known for years and leads to the accumulation of aroma compounds from the lactone group, mainly γ-decalactones [60,61,62]. The main pathway of fatty acid degradation is β-oxidation, which occurs in peroxisomes. It consists of a cycle of four reactions, as a result of which the carbon chain is shortened by two carbon units each time. Finally, 4-hydroxy-decanoyl-CoA is formed from ricinoleic acid, which spontaneously cyclizes to GDL. The esterification of 4-hydroxy-decanoic acid occurs competitively to β-oxidation at the C10 level [63]. Oxygen availability is a key parameter affecting the degree of oxidation and bioconversion of ricinoleic acid (C18) to shorter-chain β-oxidation intermediates: 3-hydroxy-γ-decalactone, dec-2-en-4-olide and dec-3-en-4-olide [29]. According to Aguedo et al. [63], the activities of enzymes such as acyl-CoA oxidase and 3-hydroxyacyl-CoA dehydrogenase are regulated by the availability of oxygen, which is necessary for the regeneration of FAD^+^ and, more indirectly, NAD^+^ cofactors.

The study has shown (Table 3) that in media based on air-treated plasma water or Nates plasma water, the concentration of 4-hydroxydecanoic acid (a precursor of GDL), as well as other γ-decalactones, was higher than the control or PWTN medium by about 2.5–4% after 72 h of cultivation (at the time when the profile of volatile compounds in the media was analyzed). This would indicate a more efficient β-oxidation cycle of ricinoleic acid in the presence of a higher concentration of oxygen in the medium (both waters used in the media contained higher concentrations of oxygen, including singlet oxygen, due to plasma treated in the presence of oxygen or air). Higher lactone concentrations of 3-hydroxy-gamma-decalactone as well as decene-4-olides were also detected in the volatile fraction of these media. The presence of the latter confirms that β-oxidation in *Y. lipolytica* yeast follows established pathways, but at the C10 level, when the hydroxy group is at the γ-carbon, there is competition between the next β-oxidation reaction and the hydrolysis of the CoA-ester or lactonization. According to Try et al. [29] in aerobic cultures, the oxygen present in the medium is first directed to long-chain catabolism. The synthesis of other lactones is affected by beta-oxidation at the C10 level and the activity of enzymes such as acyl-CoA oxidase, enoyl-CoA hydratase, 3-hydroxy-acyl-CoA dehydrogenase or keto-acyl-CoA thiolase. The presence of 3-hydroxy-γ-decalactone and decen-4-olides in the analyzed samples confirms the activity of the first two mentioned enzymes regardless of the type of medium. The lower concentration of lactones in media with a lower content of dissolved oxygen (control medium and PWTN) confirms the role of this gas in the activity of the β-oxidation cycle enzymes at the C10 level. The literature data [29,60,61,63] suggest that the availability of oxygen increases the activity of acyl-CoA oxidase and decreases the activity of 3-hydroxy-acyl-CoA dehydrogenase, resulting in high yields of 3-hydroxy-γ-decalactone and lower concentrations of GDL. This would explain the higher concentrations of this compound and dec-3-en-4-olide in media with higher oxygen concentrations (PWTA and Nantes PW).

## 4. Materials and Methods

### 4.1. Microorganism and Preculture

*Y. lipolytica* KKP 379 was obtained from the Collection of Industrial Microorganisms at the Prof. Wacław Dąbrowski Institute of Agricultural and Food Biotechnology (Warsaw, Poland). The yeast was stored in a −20 °C freezer in 20% (*v*/*v*) glycerol until used. Yeast cells were prepared by spreading 10 µL with a microbiological loop onto a regular YPG agar plate (Yeast extract 10 g/L, Peptone 20 g/L, Glucose 20 g/L—BTL Łódź, Poland) and incubating for 48 h at 28 °C.

### 4.2. Preparation of Plasma Water

#### 4.2.1. Water Treated with LPGP in the Air

Distilled water (200 mL) in 250 mL polyethylene bottles was placed in the chamber of the reactor [14] and exposed to plasma for 30 min. The plasma of 38 °C was generated in the lamp at 5 × 10^−3^ mbar, 600 V, 50 mA and 10 KHz frequency. The chamber with the water sample remained at normal pressure. The produced water was stored at ambient temperature in 1 L closed Teflon containers.

#### 4.2.2. Water Treated with LPGP under Nitrogen

Through the distilled water, a stream of nitrogen was bubbled for 15 min with a flow rate of 10 mL/min. The nitrogen was deoxygenated by passing through an absorber filled with an alkaline solution of resorcinol. After placing the bottles in plasmothrone, its chamber and the free space over the water were additionally filled with deoxygenated nitrogen. The conditions of treatment water with LPGP are the same as described in Section 4.2.1.

#### 4.2.3. Nantes Plasma Water

Nantes plasma water is a commercially available product of the Nantes Systemy Nanotechnologii company from Bolesławiec (Poland) with the following composition declared: NH_4_^+^ < 0.05 mg/L; NO_3_^−^ 28.3 mg/L; NO_2_^−^ < 0.08 mg/L; Fe < 50 µg/L; Mn 6 µg/L; water hardness 137 mg CaCO_3_/L; oxygen 8.13 mg/L; singlet oxygen 10–12 mg/L; pH 7.5.

### 4.3. Inoculation Culture

Inoculation cultures of *Y. lipolytica* KKP 379 were performed in Erlenmeyer flasks with a volume of 250 mL (working volume of 50 mL) at a temperature of 28 °C with shaking at the level of 140 rpm. After 24 h of cultivation, the inoculum was transferred into biotransformation media at an initial concentration OD_600_ = 0.25.

### 4.4. Biotransformation Condition

Biotransformation cultures of *Y. lipolytica* KKP 379 were carried out in Erlenmeyer flasks of a total volume of 500 mL. Biotransformation media (working volume 100 mL) were prepared on the basis of one of the tested plasma waters (low-pressure glow plasma water treated in air, under nitrogen or Nantes plasma water with increased content of singlet oxygen. Distilled non-treated water was used in the control medium. Biotransformation media contained 100 g/L of castor oil, 2 g/L of Tween 20 and 20 g/L peptone and were homogenized for 3 min using an IKA T25 Digital homogenizer (10,000 rpm) (Königswinter, Germany). The cultures were carried out for a maximum of 8 days at 28 °C with shaking at 140 rpm. The biotransformation was performed in triplicate for each variant of plasma water.

### 4.5. Growth Parameters

For measuring the optical density, 1 mL of culture was centrifuged using an Eppendorf microcentrifuge (5418, ROTH). The supernatant was discarded, and the cells were suspended in 1 mL of distilled water. Samples were diluted (OD between 0.7 and 1.3) and measured with a spectrophotometer UV-140 VIS THERMO Scientific Helios (Whaltam, MA, USA) at 600 nm (OD_600_); then, the obtained value of the optical density was corrected with the dilution factor.

The biomass yield was evaluated by cell dry mass measured by the thermogravimetric method. Yeast cells were separated from the culture medium by centrifugation (8000× *g*, 10 min; centrifuge MPW-351R) and then washed in a mixture of acetone/ethanol (1:1, *v*/*v*) followed by another washing with distilled water and oven-dried at 105 °C until constant weight. The results of biomass yield were given with respect to 1 L of medium (g_d.w._ of cells/L).

### 4.6. Freeze-Drying and Purification of Yeast Lipase

To obtain the active yeast lipase fraction YLLip2, after 48 h cultivation of *Y. lipolytica*, the crude enzyme solution of extracellular lipases was separated from biomass. The obtained supernatant was poured into Petri dishes and then lyophilized. The samples were frozen in an Irinox freezer (Corbanese, Italy) at −40 °C and then freeze-dried in the Christ Gamma 1–16 apparatus (Osterode am Harz, Germany). The lyophilized supernatant was purified by using ion-exchange chromatography (elution with a linear gradient with 0.7 M NaCl + 15 mM Tris-HCl, pH = 6.8, TRIS buffer) and molecular sieves (50 mM phosphate buffer, pH = 7.0). The obtained active fractions were concentrated in a centrifuge (4000× *g*, 10 min) on a VIVASPIN Centrifugal Concentrator Membrane 10.000 MWCO PES (Sartorius, Göttingen, Germany).

### 4.7. Determination of Extracellular Lipase Activity

The extracellular lipase activity of *Y. lipolytica* was determined by a spectrophotometric method based on the hydrolysis of *p*-nitrophenyl laurate [64]. For the purified YLlip2 fraction, other *p*-nitrophenyl esters (octanoate, stearate and oleate) were also used. The reaction was carried out in Eppendorf test tubes. First, 100 µL of free liquid lipase was stirred at 37 °C with 25 µL of 0.3 mmol *p*-nitrophenyl ester dissolved in 2 mL of heptane. After 15 min of incubation, absorbance was measured at 410 nm with a UV-Vis spectrophotometer. One unit (1 U) of lipase activity was defined as the amount of enzyme able to release 1 µmol of *p*-nitrophenol in 1 min at 37 °C under the conditions of the assay.

### 4.8. Determination of Acid Value

In order to determine the acid number of the lipid fraction of the media, the supernatant (after cell centrifugation) from the individual microbiological cultures was extracted 2 times in a ratio of 1:0.5 with methylene chloride. The organic phase was dried with anhydrous magnesium sulfate, and the solvent was evaporated. The obtained samples of the lipid fraction were used to determine the acid value.

The acid value of the samples (AVs) was determined by titration with 0.1 M KOH, using the following equation:

AVs = 56.11 × (V_s_ − V_b_) × F/m (1)

where AVs—acid value (mg of KOH/g of sample); V_s_—titration volume of sample (mL); V_b_—titration volume of blank (mL); F—KOH solution correction factor; m—mass of sample (g).

The acid value was determined as the number of milligrams of potassium hydroxide required to neutralize the free fatty acids in 1.0 g of oil.

### 4.9. Extraction and Quantification of Gamma-Decalactone

Gamma-decalactone was extracted from 2 mL of medium. To stop the activity of enzymes and to achieve the complete lactonization of 4-hydroxydecanoic acid, 10 µL (1 M) of HCl was added to the samples. γ-undecalactone (≥98%, Sigma-Aldrich, St. Louis, MO, USA) was introduced as an internal standard. The detailed extraction and analysis method of the organic phase by GC was described in previous works [64].

### 4.10. Separation and Identification of the Components of the Volatile Supernatant Fraction

Separation and identification of the extracted components of the volatile supernatant fraction were carried out in a gas chromatograph coupled with a mass spectrometer GCMS-QP2020 NX, SHIMADZU using the HS-SPME-GC-MS technique. The gas chromatograph was equipped with a ZB-5ms capillary column (length 30.0 m, internal diameter 0.25 mm, film thickness 0.25 μm). Volatile compounds were extracted by microextraction from the headspace (three-phase fiber, stationary phase DVB/CAR/PDMS—Divinylbenzene/Carboxen/Polydimethylsiloxan, Supelco, Phenomenex).

The temperature of the injector was 230 °C. A stream divider of 1:30 was used. The carrier gas (helium) flow rate in the column was 1.50 mL/min. The gas chromatograph oven temperature was held at 45 °C for 5 min. The temperature was then increased at a rate of 5 °C/min to 195 °C and then at a rate of 25 °C/min to 270 °C, which was held for 5 min. The mass spectrometer was operated in the TIC mode in the 40–400 mass range. The junction line temperature was 200 °C. The temperature of the ion source was 200 °C. The identification of the volatile compounds was completed by comparing the retention indexes of the compounds data with literature and databases (http://www.pherobase.com/database/kovats/kovats-index.php (accessed on 15 June 2023), http://www.odour.org.uk/lriindex.html (accessed on 15 June 2023)) and mass spectra with NIST 11, NIST 11s libraries.

The content of volatile compounds was presented as a percentage of all compounds in the volatile fraction and the content of individual compounds in relation to the internal standard (*n*-propylbenzol).

The preparation of samples for determination consisted of collecting a 4 mL sample into a 20 mL chromatographic vial. The vial was sealed with an aluminum cap. Fiber conditioning was applied for 30 min at 270 °C in the injector port. The sample was kept at 45 °C for 20 min, and then the volatiles were extracted by SPME at 45 °C for another 20 min. Fiber desorption took place in the injector port at 230 °C for 5 min. Each sample was analyzed in 2 replicates.

### 4.11. Statistical Analysis

The obtained results were analyzed by statistical methods, using the STATISTICA 13.1 software (Tibco Software, CA, USA). To determine the significance of the grouping variables, a one-way ANOVA with Tukey’s HSD test was used. Values of *p* ≤ 0.05 were considered to be statistically significant.

## 5. Conclusions

Undoubtedly searching for novel approaches in increasing the biotransformation yield performed by living cells is worth the effort and has a great potential in green chemistry. This study demonstrates an example of the use of cold plasma technology for plasma water produce, which is used in microbiological media. Low-temperature plasma processing is a cutting-edge technology driving innovation in the bioindustry. Plasma water, thanks to its unique physicochemical properties, including macrostructure modifications, can have a beneficial effect on microorganisms.

Few aspects have been investigated including the impact of some variants of cold plasma-based culture media on yeast cell growth, selected enzymes activity and biotransformation final product yield. Notably, it was shown that plasma water with an increased content of singlet oxygen can stand as a good solvent for media ingredients in order to achieve high biomass concentration in cultures of aerobic microorganisms. For *Y. lipolytica* yeast, an increased content of oxygen in the medium did not affect extracellular lipase activity. This leads to the conclusion that the general metabolic condition of the cells grown in low-pressure glow plasma water saturated with molecular oxygen was better than those cells grown in plasma saturated with air or nitrogen. Therefore, lipid hydrolysis (castor oil to ricinoleic acid) was enhanced, but this step seems not to be crucial for GDL concentration. Interestingly, the research confirmed an important role of oxygen of the β-oxidation enzymes at the C10 level and finally in the bioconversion of ricinoleic acid to GDL and other lactones. This study suggests that media based on water with heightened oxygen levels exhibit higher efficiency of the synthesis of gamma-declacatones.

As a note, future research should be more interdisciplinary and focused on the biophysical properties of oxygen in culture media. Monitoring oxygen species in the medium and their connection with enzyme activity (studied at the genetic and phenotypic level) may prove to be particularly interesting from the point of view of the biosynthetic pathways of various lactones using biotechnological methods, and it will also allow the development of the possibility of using plasma-treated water.

## Figures and Tables

**Table 1:** The maximum specific growth rate (μ_max_), maximum biomass concentration (X_max_, g/L/10^8^ cells) and the level of yield of biomass from the substrate (YX_max_/S, g_d.w._/g) of *Y. lipolytica* cultivated in medium based on low-pressure glow plasma water saturated with air, nitrogen or molecular oxygen (Nantes PW). Kinetic parameters were calculated according to Try et al. [29] and Małajowicz et al. [30].

Parameters	Type of Medium			
	Control Medium	PWTN	PWTA	Nantes PW
µ_max_ (h^−1^)	0.056 ^a^	0.088 ^b^	0.062 ^a^	0.136 ^c^
X_max_ (g of dried mass/10^8^ cells)	1.41 ^a^	1.74 ^b^	1.60 ^b^	2.29 ^c^
YX_max_/S (g_d.w._/g)	0.066 ^a^	0.105 ^c^	0.087 ^b^	0.163 ^d^

Mean values followed by the same letter are not significantly different according to HSD Tukey (*p* ≤ 0.05).

**Table 2:** Kinetic parameters of GDL biosynthesis by *Y. lipolytica* cultivated in media based on water treated with low-temperature, low-pressure glow plasma in contact with air (PWTA) or nitrogen (PWTN) and Nantes plasma water with heightened singlet oxygen level. Kinetic parameters were calculated according to Małajowicz et al. [30].

Parameters	Type of Medium			
	Control Medium	PWTN	PWTA	Nantes PW
Y_GDL_/X_max_ (g/g_d.w._)	0.359 ^a^	0.334 ^b^	0.358 ^a^	0.294 ^c^
Y_GDL_/S (g/g)	0.028 ^a^	0,031 ^a^	0.031 ^a^	0.048 ^b^
qLV_GDL_ (g/L/h)	0.023 ^a^	0.029 ^b^	0.026 ^a^	0.033 ^c^
qL_GDL_ (g/g_d.w._/h)	0.0030 ^a^	0.0028 ^b^	0.0030 ^a^	0.0020 ^c^

Mean values followed by the same letter are not significantly different according to HSD Tukey (*p* ≤ 0.05). Y_GDL_/X_max_—conversion of DGL per biomass yield. Y_GDL_/S—conversion of GDL per carbon substrate. qLV_GDL_—volumetric rate of gamma-decalactone production. qL_GDL_—specific rate of gamma-decalactone production.

**Table 3:** Volatile compounds (percentage content) detected in biotransformation media (after 72 h) based on water treated with low-temperature, low-pressure glow plasma in contact with air (PWTA), nitrogen (PWTN) or Nantes plasma water with heightened singlet oxygen level.

Compounds	Type of Medium			
	Control Medium	PWTN	PWTA	Nantes PW
	**Percentage Content (%)**			
**Higher alcohols**	15.05 ^c^ ± 0.23	8.92 ^b^ ± 0.97	7.33 ^a^ ± 0.21	7.53 ^a^ ± 0.69
3-methyl-butan-1-ol				
*n*-heptanol				
oct-1-en-3-ol				
2-phenylethanol				
**Aldehydes**	39.59 ^a^ ± 0.86	44.47 ^b^ ± 3.15	41.33 ^a^ ± 0.77	40.28 ^a^ ± 3.53
hexanal				
heptanal				
benzaldehyde				
phenylacetaldehyde				
**Ketones**	7.17 ^a^ ± 0.18	7.54 ^a^ ± 0.54	7.21 ^a^ ± 0.11	7.03 ^a^ ± 0.12
2-octanone				
3-methylene-2-pentanone				
**Fatty acids**	15.43 ^b^ ± 0.14	11.82 ^a^ ± 0.57	12.31 ^a^ ± 0.22	11.38 ^a^ ± 0.09
2-methyl-butanoic acid				
3-methyl-butanoic acid				
2-hydroxyoctanoic acid				
4-hydroxydecanoic acid				
3,4-dihydroxy-decanoic acid				
**Lactones**	13.01 ^a^ ± 0.44	14.89 ^a^ ± 0.52	22.03 ^b^ ± 0.87	24.55 ^b^ ± 0.36
gamma-butyrolactone				
gamma-nonalactone				
gamma-decalactone				
3-hydroxy-gamma-decalactone				
dec-2-en-4-olide				
dec-3-en-4-olide				
**Others**	9.75 ^a^ ± 0.77	12.36 ^b^ ± 1.98	9.79 ^a^ ± 0.92	9.23 ^a^ ± 1.07
2,2-dimethylhexane				
2,4-dimethylhexane				
2,5-dimethylhexane				
3-ethylpentane				
*o*-xylene				
2-heptylfuran				
3-metyhyl-undec-5-ene				
1-dodecene				

Mean values followed by the same letter are not significantly different according to HSD Tukey (*p* ≤ 0.05). Difference in colors presented indicate differences in content of compounds in the samples: yellow (<3%), blue (3–5%), red (≥5%).

## Data Availability

The data presented in this study are available on request from the corresponding author (J.M.).
